# Interpretation of coefficients in segmented regression for interrupted time series analyses

**DOI:** 10.21203/rs.3.rs-3972428/v1

**Published:** 2024-02-27

**Authors:** Yongzhe Wang, Narissa J. Nonzee, Haonan Zhang, Kimlin T. Ashing, Gaole Song, Catherine M. Crespi

**Affiliations:** City Of Hope National Medical Center; City Of Hope National Medical Center; University of Washington; City Of Hope National Medical Center; City Of Hope National Medical Center; University of California, Los Angeles

**Keywords:** observational study, interrupted time series design, segmented regression, healthcare policy evaluation, coefficient interpretation

## Abstract

**Background:**

Segmented regression, a common model for interrupted time series (ITS) analysis, primarily utilizes two equation parametrizations. Interpretations of coefficients vary between the two segmented regression parametrizations, leading to occasional user misinterpretations.

**Methods:**

To illustrate differences in coefficient interpretation between two common parametrizations of segmented regression in ITS analysis, we derived analytical results and present an illustration evaluating the impact of a smoking regulation policy in Italy using a publicly accessible dataset. Estimated coefficients and their standard errors were obtained using two commonly used parametrizations for segmented regression with continuous outcomes. We clarified coefficient interpretations and intervention effect calculations.

**Results:**

Our investigation revealed that both parametrizations represent the same model. However, due to differences in parametrization, the immediate effect of the intervention is estimated differently under the two approaches. The key difference lies in the interpretation of the coefficient related to the binary indicator for intervention implementation, impacting the calculation of the immediate effect.

**Conclusions:**

Two common parametrizations of segmented regression represent the same model but have different interpretations of a key coefficient. Researchers employing either parametrization should exercise caution when interpreting coefficients and calculating intervention effects.

## Background

The interrupted time series (ITS) design is an increasingly popular quasi-experimental design that is used to estimate the effectiveness of an intervention when a randomized trial is not feasible.(1–7) In an ITS design, observations are collected in a time series over a study period that includes intervals both before and after the introduction of an intervention, and these observations are contrasted to estimate the intervention’s effectiveness. ITS designs have been used widely in health services research, for example, in the evaluation of health policies and health care quality improvement interventions in real-world settings. (2, 8–14)

The most widely used method of analyzing data from an ITS design study is segmented regression.(1, 2, 4–6, 15, 16) Segmented regression, also known as piecewise regression or broken-stick regression, is a method in regression analysis in which a series of observations is partitioned into intervals and a separate line segment is fit to each interval. The theoretical framework for estimating segmented regression dates back to the work of Quandt.(17, 18) The use of segmented regression for ITS dates back to its application in evaluating cross-sectional time series experiments in psychology.(19)

There are two common parametrizations for segmented regression applied to ITS analyses, that of Bernal et al.(6, 7) and that of Wagner et al.(4) Superficially, these two parametrizations appear similar, but they have important differences that impact the estimation of intervention effects, raising concerns about the potential for misinterpretation of results.(20) This paper investigates the two different parametrizations and their interpretations and illustrates the differences in interpretation by applying them to a real data set. (7)

## Methods

### Parametrizations of Segmented Regression

To explain the two common parametrizations of segmented regression for ITS, we consider the setting of a single interrupted time series collected from one unit (for example, a single clinic) with a continuous outcome variable.(3, 7) The key features of the model equation are a variable for continuous time, a binary indicator denoting the presence of an intervention, and an outcome measure.(1–3, 6, 7, 14, 15, 21) Let T represent continuous time measuring the duration since the study’s initiation, starting from 0, and let δ denote the time at which the intervention is introduced. $X_{t}$ represents a binary indicator denoting the presence or absence of an intervention at time t, equal to 0 for T<δ and 1 for T≥δ. Let $y_{t}$ denote the continuous outcome as measured at time t.

Bernal’s parametrization involves regressing the outcome $y_{t}$ on $T,X_{t}$, and their interaction.(6, 7, 19, 22–25) Bernal’s parametrization(7) is:

(2) $$y_{t}=\beta_{0}+\beta_{1}T+\beta_{2}^{B}X_{t}+\beta_{3}X_{t}T(1)=\left\{\begin{array}{c} \beta_{0}+\beta_{1}T,T<\delta \\ \left(\beta_{0}+\beta_{2}^{B}\right)+\left(\beta_{1}+\beta_{3}\right)T,T\geq \delta \end{array}\right.$$

In this parametrization, $\beta_{0}$ is the intercept in the pre-intervention interval and represents the mean outcome level at the inception of the study $(T=0).\beta_{1}$ is the slope during the pre-intervention interval and represents the mean change in the outcome for a one unit increase in time. For the post-intervention interval, $\beta_{0}+\beta_{2}^{B}$ is the intercept and $\beta_{1}+\beta_{3}$ is the slope. Note that $\beta_{0}+\beta_{2}^{B}$ represents the outcome level at time 0 if we extrapolated the post-intervention regression line backwards in time. The coefficients $\beta_{2}^{B}$ and $\beta_{3}$ represent the differences in intercept and slope between the pre- and post-intervention intervals. Thus, this model allows for different linear regression models (different intercepts and different slopes) during the pre-and post-intervention intervals.

Two different aspects of an intervention effect can be captured with this segmented regression model.(4, 5, 21, 26, 27) One aspect is a change in the mean level of the outcome at time δ, corresponding to an immediate effect of the intervention on the outcome. The other aspect is the change in slopes from pre- to post-intervention, which represents a longer-term, gradual effect of the intervention on the outcome. In Bernal’s parametrization, the gradual effect corresponds to the change in slopes, which is $\beta_{3}$ in Eq. (1). However, the immediate effect does not correspond to the difference in intercepts $\left(\beta_{2}^{B}\right)$.(4, 28) Rather, the immediate effect is the difference in means between the pre- and post-intervention models at the start of the intervention at time δ, which can be formulated as:

$$\text{ChangeinLevels}\;=\left(\beta_{0}+\beta_{2}^{B}\right)+\left(\beta_{1}+\beta_{3}\right)\delta -\left(\beta_{0}+\beta_{1}\delta \right)=\beta_{2}^{B}+\beta_{3}\delta .$$

Hence in Bernal’s parametrization, $\beta_{2}^{B}$ is the difference in intercepts between the pre-and post-intervention models, that is, the vertical difference between the two regression lines at time 0, and the immediate effect is given by $\beta_{2}^{B}+\beta_{3}\delta$.

The parametrization of segmented regression advanced by Wagner is the same as Bernal’s parametrization except for the interaction term.(4) In Wagner’s parametrization, the interaction is the product of the binary intervention indicator and the time elapsed since the intervention’s implementation, T-δ. The model is:

(4) $$y_{t}=\beta_{0}+\beta_{1}T+\beta_{2}^{W}X_{t}+\beta_{3}X_{t}(T-\delta )(3)=\left\{\begin{array}{c} \beta_{0}+\beta_{1}T,T<\delta \\ \left(\beta_{0}+\beta_{2}^{W}-\beta_{3}\delta \right)+\left(\beta_{1}+\beta_{3}\right)T,T\geq \delta \end{array}\right.$$

Under this parametrization, the intercept and slope of the pre-intervention model are the same as for Bernal, but the intercept and slope of the post-intervention model are $\beta_{0}+\beta_{2}^{W}-\beta_{3}\delta$ and $\beta_{1}+\beta_{3}$, respectively. Thus, the two parametrizations differ in the parametrization of the intercept of the postintervention model. The difference in intercepts between the pre- and post-intervention models is $\beta_{2}^{W}-\beta_{3}\delta$. For intervention effects, $\beta_{3}$ represents the gradual effect, as it does in Bernal’s parametrization. However, the immediate effect, quantified as the mean change in levels at time δ, is given by:

$$\text{ChangeinLevels}\;=\left(\beta_{0}+\beta_{2}^{W}-\beta_{3}\delta \right)+\left(\beta_{1}+\beta_{3}\right)\delta -\left(\beta_{0}+\beta_{1}\delta \right)=\beta_{2}^{W}.$$

Consequently, in this parametrization, $\beta_{2}^{W}$ captures the difference in means at the start of the intervention’s implementation. Thus when researchers use Wagner’s parametrization, the immediate effect can be directly extracted from $\beta_{2}^{W}$.

It is important to highlight that the intercept and slope coefficients for the pre-intervention models in both parametrizations are the same. Additionally, the post-intervention slopes are the same, being represented by $\beta_{1}+\beta_{3}$ in both equations (2) and (4). The intercept terms of the two parametrizations are different: $\beta_{0}+\beta_{2}^{B}$ in Eq. (2) and $\beta_{0}+\beta_{2}^{W}-\beta_{3}\delta$ in equation(4) . Assuming the post-intervention intercepts under the two parametrizations are equivalent, we can find that:

$$\beta_{0}+\beta_{2}^{B}=\beta_{0}+\beta_{2}^{W}-\beta_{3}\delta \beta_{2}^{W}=\beta_{2}^{B}+\beta_{3}\delta$$

Hence, despite the differences between the two parametrizations, they should give the same estimate of the immediate effect of the intervention. In the next section, we show the alignment between the two parametrizations through the analytical expressions of the estimated coefficients. We summarize the interpretation of coefficients and intervention effects under the two different parametrizations in Table 1.

### Estimated Coefficients

As observed, the parametrizations of segmented regression proposed by Wagner et al. and Bernal et al. have different model equations but correspond to the same pre- and post-intervention models. The two parametrizations also lead to different design matrices. The design matrix for Bernal’s parametrization is

$$X_{B}=\left[\begin{array}{cccc} 1 & t_{1} & 0 & 0\\ 1 & t_{2} & 0 & 0\\ 1 & t_{3} & 0 & 0\\ \vdots & \vdots & \vdots & \vdots \\ 1 & t_{m} & 0 & 0\\ 1 & t_{m+1} & 1 & t_{m+1}\\ 1 & t_{m+2} & 1 & t_{m+2}\\ \vdots & \vdots & \vdots & \vdots \\ 1 & t_{m+n} & 1 & t_{m+n} \end{array}\right]$$

where the upper part of the matrix represents the pre-intervention period, and the lower part represents the post-intervention period. We assume that there are m and n observations in the pre- and post-intervention periods, respectively, for a total of N=m+n observations. The design matrix for Wagner’s parametrization is

$$X_{W}=\left[\begin{array}{cccc} 1 & t_{1} & 0 & 0\\ 1 & t_{2} & 0 & 0\\ 1 & t_{3} & 0 & 0\\ \vdots & \vdots & \vdots & \vdots \\ 1 & t_{m} & 0 & 0\\ 1 & t_{m+1} & 1 & t_{m+1}-\delta \\ 1 & t_{m+2} & 1 & t_{m+2}-\delta \\ \vdots & \vdots & \vdots & \vdots \\ 1 & t_{m+n} & 1 & t_{m+n}-\delta \end{array}\right]$$

Using design matrices $X_{B}$ or $X_{W}$, we can obtain the ordinary least squares estimates of regression coefficients $\setminus \text{varvec}\beta =\left[\beta_{0},\beta_{1},\beta_{2},\beta_{3}\right]\prime$ by solving the normal equations, obtaining $\hat{\text{varvec}}\beta ={\left(X^{\setminus \text{varvec}T}X\right)}^{-1}X^{\setminus \text{varvec}T}y$ where $y=\left[y_{1},y_{2},\cdots ,y_{m},y_{m+1},\cdots ,y_{m+n}\right]'$is the vector of the outcome variable. The covariance matrix for∖varvec⁡β can be obtained as $\hat{\text{varvec}}\Sigma ={\hat{\sigma }}^{2}{\left(X^{\setminus \text{varvec}T}X\right)}^{-1}$ where ${\hat{\sigma }}^{2}$ represents the estimated residual, calculated as ${\hat{\sigma }}^{2}=\frac{1}{N-p}(y-X\setminus \hat{\text{varvec}}\beta {)}^{T}(y-X\setminus \hat{\text{varvec}}\beta )$ where p indicates the number of columns in the design matrix. We will show the estimates of ∖varvec⁡β and ∖varvec⁡Σ in ordinary algebra rather than matrix algebra.


$$\setminus \text{varvec}\beta_{0},\setminus \text{varvec}\beta_{1},\text{and}\;\setminus \text{varvec}\beta_{3}$$

The estimates of $\beta_{0},\beta_{1}$, and $\beta_{3}$ take the forms

$${\hat{\beta }}_{0}=\frac{\left(\sum_{j=1}^{m}\;t_{j}y_{j}\right)\left(\sum_{j=1}^{m}\;t_{j}\right)-\left(\sum_{j=1}^{m}\;y_{j}\right)\left(\sum_{j=1}^{m}\;t_{j}^{2}\right)}{{\left(\sum_{j=1}^{m}\;t_{j}\right)}^{2}-m\left(\sum_{j=1}^{m}\;t_{j}^{2}\right)},{\hat{\beta }}_{1}=\frac{\left(\sum_{j=1}^{m}\;t_{j}\right)\left(\sum_{j=1}^{m}\;y_{j}\right)-m\left(\sum_{j=1}^{m}\;t_{j}y_{j}\right)}{{\left(\sum_{j=1}^{m}\;t_{j}\right)}^{2}-m\left(\sum_{j=1}^{m}\;t_{j}^{2}\right)},\;{\hat{\beta }}_{3}=\frac{\left(\sum_{j=m+1}^{m+n}\;t_{j}\right)\left(\sum_{j=m+1}^{m+n}\;y_{j}\right)-n\left(\sum_{j=m+1}^{m+n}\;t_{j}y_{j}\right)}{{\left(\sum_{j=m+1}^{m+n}\;t_{j}\right)}^{2}-n\left(\sum_{j=m+1}^{m+n}\;t_{j}^{2}\right)}-{\hat{\beta }}_{1},={\hat{\beta }}_{3,\text{post}}-{\hat{\beta }}_{1},$$

where ${\hat{\beta }}_{3,\text{post}}$ represents the post-intervention slope such that ${\hat{\beta }}_{3,\text{post}}={\hat{\beta }}_{1}+{\hat{\beta }}_{3}$. The summations j=1 to m and j=m+1 to m+n represent the summation over observations from the pre- and postintervention periods, respectively. Under both parametrizations, ${\hat{\beta }}_{0}$ represents the mean outcome at study initiation and serves as the intercept in the pre-intervention model, ${\hat{\beta }}_{1}$ represents the pre-intervention slope, and ${\hat{\beta }}_{3}$ represents the difference in slopes between the pre-and post-intervention models. Note that ${\hat{\beta }}_{0}$ and ${\hat{\beta }}_{1}$ use only information from the pre-intervention period while ${\hat{\beta }}_{3}$ uses observations from each period to estimate a period-specific slope and then takes the difference. The estimated variances of these coefficients are

$$\text{var}\left({\hat{\beta }}_{0}\right)={\hat{\sigma }}^{2}\frac{\sum_{j=1}^{m}\;\;t_{j}^{2}}{m\sum_{j=1}^{m}\;\;{\left(t_{j}-{\overset{‾}{t}}_{m}\right)}^{2}},\text{var}\left({\hat{\beta }}_{1}\right)={\hat{\sigma }}^{2}\frac{1}{\sum_{j=1}^{m}\;\;{\left(t_{j}-{\overset{‾}{t}}_{m}\right)}^{2}},\;\text{var}\left({\hat{\beta }}_{3}\right)={\hat{\sigma }}^{2}\left[\frac{1}{\sum_{j=1}^{m}\;\;{\left(t_{j}-{\overset{‾}{t}}_{m}\right)}^{2}}+\frac{1}{\sum_{j=m+1}^{m+n}\;\;{\left(t_{j}-{\overset{‾}{t}}_{n}\right)}^{2}}\right],\;{\text{wheret}}_{m}=\frac{1}{m}\sum_{j=1}^{m}\;\;t_{j}\text{and}{\overset{‾}{t}}_{n}=\frac{1}{n}\sum_{j=m+1}^{m+n}\;\;t_{j}.\;\setminus \text{varvec}\beta_{2}$$

The estimates of $\beta_{2}$ values for the two different parametrizations are:

$${\hat{\beta }}_{2}^{B}=\frac{\left(\sum_{j=m+1}^{m+n}\;t_{j}y_{j}\right)\left(\sum_{j=m+1}^{m+n}\;t_{j}\right)-\left(\sum_{j=m+1}^{m+n}\;y_{j}\right)\left(\sum_{j=m+1}^{m+n}\;t_{j}^{2}\right)}{{\left(\sum_{j=m+1}^{m+n}\;t_{j}\right)}^{2}-n\left(\sum_{j=m+1}^{m+n}\;t_{j}^{2}\right)}-{\hat{\beta }}_{0}={\hat{\beta }}_{2,\text{post}}^{B}-{\hat{\beta }}_{0},\;{\hat{\beta }}_{2}^{W}={\hat{\beta }}_{2}^{B}+\delta {\hat{\beta }}_{3}=\left({\hat{\beta }}_{2,\text{post }}^{B}+\delta {\hat{\beta }}_{3,\text{ post }}\right)-\left({\hat{\beta }}_{0}+\delta {\hat{\beta }}_{1}\right),$$

where ${\hat{\beta }}_{2,\text{post}}^{B}$ represents the post-intervention intercept under Bernal’s parametrization such that ${\hat{\beta }}_{2,\text{post}}^{B}={\hat{\beta }}_{0}+{\hat{\beta }}_{2}^{B}\cdot {\hat{\beta }}_{2}^{B}$ corresponds to the difference in intercepts between the pre- and post-intervention models. On the other hand, ${\hat{\beta }}_{2}^{W}$ corresponds to the difference in the mean outcome at the time of intervention implementation. The estimated variances for ${\hat{\beta }}_{2}$ for the two parametrizations are

$$\text{var}\left({\hat{\beta }}_{2}^{B}\right)={\hat{\sigma }}^{2}\left[\frac{\sum_{j=1}^{m}\;t_{j}^{2}}{m\sum_{j=1}^{m}\;{\left(t_{j}-{\overset{‾}{t}}_{m}\right)}^{2}}+\frac{\sum_{j=m+1}^{m+n}\;t_{j}^{2}}{n\sum_{j=m+1}^{m+n}\;{\left(t_{j}-{\overset{‾}{t}}_{n}\right)}^{2}}\right],\;\text{var}\left({\hat{\beta }}_{2}^{W}\right)={\hat{\sigma }}^{2}\left[\frac{\sum_{j=1}^{m}\;{\left(t_{j}-\delta \right)}^{2}}{m\sum_{j=1}^{m}\;{\left(t_{j}-{\overset{‾}{t}}_{m}\right)}^{2}}+\frac{\sum_{j=m+1}^{m+n}\;{\left(t_{j}-\delta \right)}^{2}}{n\sum_{j=m+1}^{m+n}\;{\left(t_{j}-{\overset{‾}{t}}_{n}\right)}^{2}}\right].$$

Standard errors are obtained as the square root of the variances. For estimates of linear combinations of coefficients, such as $\beta_{2}^{B}+\beta_{3}\delta$ and $\beta_{2}^{W}-\beta_{3}\delta$, the covariance between $\beta_{2}$ and $\beta_{3}$ is also needed to obtain the standard error. We omit this formula. All standard errors can be calculated in standard software.

## Results

### Illustration

We illustrate the differences in the two parametrizations using a dataset provided by Barone-Adesi et al. (29) and analyzed by Bernal et al.(7) The objective of Bernal et al.’s study was to assess the effectiveness of a policy that banned smoking in all indoor public places in Sicily, Italy. The policy implementation began in January 2005. The researchers adopted an ITS design and collected data between 2002 and 2006 on the standardized rates of acute coronary episodes (ACE) in Sicily per month. The standardized ACE rates were computed by dividing the monthly frequency of ACE hospital admissions in Sicily by the agestandardized population per person-year. We expressed the outcome as standardized ACE rates per 1000. There were 36 and 22 observations of standardized ACE rates in the pre- and post-intervention periods, respectively. Our focus is on illustrating the two parametrizations rather than providing a detailed analysis of these data, as was done by Bernal et al.(7). Hence, we do not present a complete analysis.

**Figure 1:**
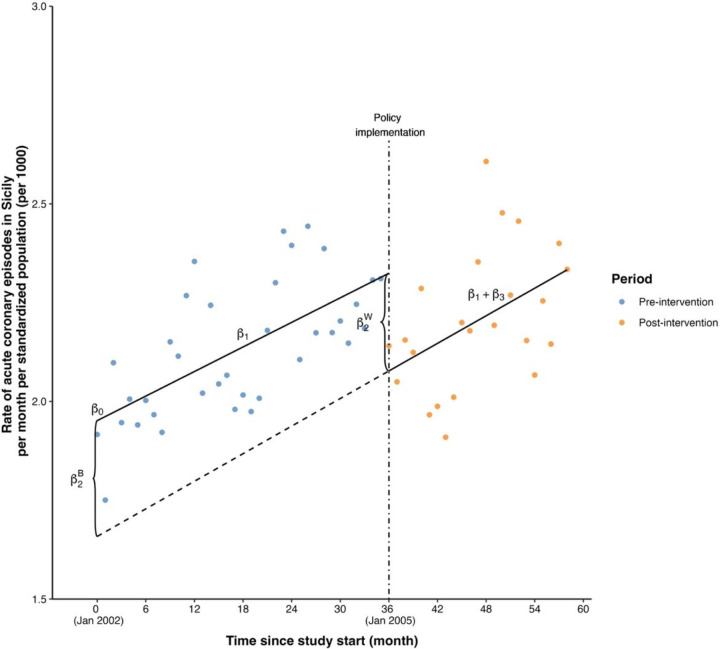
Estimated segmented regression line using the illustration dataset. The scatter points in blue and orange represented the data points in the pre- and post-intervention periods, respectively, while the black lines represented the pre- and post-intervention models. The vertical black dot-dashed line represented the time point of policy (i.e., intervention) implementation. The black dashed line represented the extension of the post-intervention model.

Table 2 displays estimated coefficients and intervention effects and standard errors calculated as described in previous sections. Figure 1 displays the fitted model. The supplementary materials include implementation details with R code. $\beta_{0}$ is the intercept of the pre-intervention model and corresponds to the standardized rate of ACE per 1000 in January 2002, estimated as 1.95 (SE 0.05). $\beta_{1}$ is the slope of the pre-intervention model and indicates that the standardized rate of ACE per 1000 was increasing an estimated 0.01 units (SE 0.002) per month during this interval. At the time of intervention onset, it is estimated that the standardized rate of ACE per 1000 had dropped by 0.25 units (SE 0.08), corresponding to an immediate intervention effect; the decrease was statistically significant (p=0.002). Thereafter, the standardized ACE rate per 1000 continued to increase at an estimated rate of 0.01 per month (SE 0.004). The difference in slopes before and after intervention onset was not significantly different from zero, indicating no evidence of a gradual intervention effect.

The difference in estimates of $\beta_{2}$ between the two parametrizations of segmented regression is noteworthy. Figure 1 visually illustrates the difference between two estimated $\beta_{2}$ values. ${\hat{\beta }}_{2}^{W}$ corresponds to the difference in the fitted outcome value at the time of intervention onset between the pre- and postintervention models (immediate effect), represented as the vertical distance between the two regression lines at that time point. In contrast, ${\hat{\beta }}_{2}^{B}$ is the difference in intercepts between the pre- and postintervention models. In this dataset, the two quantities have similar values. This is because there is little difference in slopes between the pre- and post-intervention intervals. In data in which the two slopes are different, we would expect to see a greater difference between these two values.

## Discussion

In our investigation of the two common parametrizations of segmented regression for ITS, we verified that the coefficients for baseline outcome level, pre-intervention trend, and difference in slopes pre- and postintervention onset are the same for both parametrizations. However, the interpretation of the coefficient for the binary intervention indicator differs between the two parametrizations. Under Wagner’s parametrization, this coefficient captures the difference in mean outcome between the pre- and postintervention models at the time of intervention implementation, indicating the change-in-level or immediate effect. Under Bernal’s parametrization, this coefficient is not the immediate effect but rather captures the difference in the intercept between the pre- and post-intervention models. Unfortunately, this coefficient has sometimes been misinterpreted in the literature.(28, 30–40)

When employing Bernal’s parametrization in segmented regression, it is important to recognize that the immediate effect should be calculated as a combination of two coefficients, as we have described. Conversely, when applying Wagner’s parametrization, the coefficient associated with the binary intervention indicator can be used as an estimate of the immediate effect and to get the difference in intercepts, one needs to use a combination of two coefficients. Thus, Bernal’s parametrization is more convenient for computing the difference in intercepts, while Wagner’s parametrization is more convenient for immediate effects. Users can choose between these parametrizations to tailor their estimates. Regardless of the chosen parametrization, both approaches yield the same pre- and post-intervention models.

Both parametrizations have limitations. They both hypothesize an outcome change immediately after intervention implementation and a linear change over time both before and after the intervention implementation. However, these assumptions might not accurately represent the dynamics of an ITS study; for example, intervention effects can exhibit lagged impacts. In such cases, one can consider alternative parametrizations that incorporate delayed effects or include a transition period between preintervention and post-intervention periods.(6, 16) Numerous technical issues related to segmented regression, such as autocorrelation, seasonality, and heterogeneity, have been addressed in existing literature.(1, 2, 4, 5, 15, 16, 22) By applying segmented regression and selecting appropriate parametrizations, users can employ tailored tools to mitigate technical issues based on the specifics of their data.

## Conclusion

In conclusion, two common segmented regression parametrizations in ITS analysis represent the same model, yielding identical pre- and post-intervention models but distinct coefficient interpretations. Immediate intervention effect calculations differ between parametrizations, while gradual intervention effect calculations remain consistent. Both parametrizations for segmented regression can be employed as analytical approaches for ITS design, provided the specific nuances and interpretations of the coefficients are understood and explained.

## Figures and Tables

**Table 1:** Summary of interpretation of coefficients and intervention effects in segmented regression for interrupted time series analysis using parametrizations of Bernal et al. and Wagner et al.

	Bernal’s Parametrization	Wagner’s Parametrization
Model equation	$y_{t}=\beta_{0}+\beta_{1}T+\beta_{2}^{B}X_{t}+\beta_{3}X_{t}T$	$y_{t}=\beta_{0}+\beta_{1}T+\beta_{2}^{W}X_{t}+\beta_{3}X_{t}(T-\delta )$
Interpretations	Coefficients	
Baseline level	$\beta_{0}$	
Pre-intervention trend	$\beta_{1}$	
Difference in intercepts	$\beta_{2}^{B}$	$\beta_{2}^{W}-\beta_{3}\delta$
Immediate effect (change in levels at intervention onset)	$\beta_{2}^{B}+\beta_{3}\delta$	$\beta_{2}^{W}$
Gradual effect (change in slopes after intervention)	$\beta_{3}$	
Post-intervention trend	$\beta_{1}+\beta_{3}$	

**Table 2:** Estimated coefficients^1^ in segmented regression with standard errors (SE) and P-values.

Interpretations	Coefficients		Estimate (SE)	P-value
	Bernal’s Parametrization	Wagner’s Parametrization		
Baseline level	$\beta_{0}$		1.95 (0.05)	<0.0001
Pre-intervention trend	$\beta_{1}$		0.01 (0.002)	<0.0001
Difference in intercepts	$\beta_{2}^{B}$	$\beta_{2}^{W}-\beta_{3}\delta$	−0.29 (0.22)	0.1840
Immediate effect (change in levels at intervention onset)	$\beta_{2}^{B}+\beta_{3}\delta$	$\beta_{2}^{W}$	−0.25 (0.08)	0.0018
Gradual effect (change in slopes after intervention)	$\beta_{3}$		0.001 (0.005)	0.8012
Post-intervention trend	$\beta_{1}+\beta_{3}$		0.01 (0.004)	<0.0001

## Data Availability

All data analyzed in this study are available in the published article by Bernal et al.(7)

## Footnotes

1. The estimated coefficients were derived from segmented regression, with the model equation representing standardized ACE rates per 1000 regressed on continuous time, a binary indicator denoting the presence or absence of intervention implementation, and their interaction term.

## Abbreviations

ITS: interrupted time series
ACE: acute coronary episodes

## References

[1] Ramsay CR, Matowe L, Grilli R, Grimshaw JM, Thomas RE. Interrupted time series designs in health technology assessment: lessons from two systematic reviews of behavior change strategies. Int J Technol Assess Health Care. 2003;19(4):613–23. 15095767 10.1017/s0266462303000576

[2] Hategeka C, Ruton H, Karamouzian M, Lynd LD, Law MR. Use of interrupted time series methods in the evaluation of health system quality improvement interventions: a methodological systematic review. BMJ Global Health. 2020;5(10):e003567. 33055094 10.1136/bmjgh-2020-003567 PMC7559052

[3] Kontopantelis E, Doran T, Springate DA, Buchan I, Reeves D. Regression based quasi-experimental approach when randomisation is not an option: interrupted time series analysis. BMJ. 2015;350. 10.1136/bmj.h2750 PMC4460815 26058820

[4] Wagner AK, Soumerai SB, Zhang F, Ross-Degnan D. Segmented regression analysis of interrupted time series studies in medication use research. J Clin Pharm Ther. 2002;27(4):299–309. 12174032 10.1046/j.1365-2710.2002.00430.x

[5] Gebski V, Ellingson K, Edwards J, Jernigan J, Kleinbaum D. Modelling interrupted time series to evaluate prevention and control of infection in healthcare. Epidemiol Infect. 2012;140(12):2131–41. 22335933 10.1017/S0950268812000179 PMC9152341

[6] Bernal JL, Soumerai S, Gasparrini A. A methodological framework for model selection in interrupted time series studies. J Clin Epidemiol. 2018;103:82–91. 29885427 10.1016/j.jclinepi.2018.05.026

[7] Bernal JL, Cummins S, Gasparrini A. Interrupted time series regression for the evaluation of public health interventions: a tutorial. Int J Epidemiol. 2017;46(1):348–55. 27283160 10.1093/ije/dyw098 PMC5407170

[8] Sears JM, Haight JR, Fulton-Kehoe D, Wickizer TM, Mai J, Franklin GM. Changes in early high-risk opioid prescribing practices after policy interventions in Washington State. Health Serv Res. 2021;56(1):49–60. 33011988 10.1111/1475-6773.13564 PMC7839645

[9] Rokicki S, Steenland MW, Geiger CK, Gourevitch RA, Chen L, Martin MW, Trends in postpartum mental health care before and during COVID −19. Health Serv Res. 2022;57(6):1342–7. 36059179 10.1111/1475-6773.14051 PMC9539265

[10] Balicer RD, Hoshen M, Cohen-Stavi C, Shohat-Spitzer S, Kay C, Bitterman H, Sustained Reduction in Health Disparities Achieved through Targeted Quality Improvement: One-Year Follow-up on a Three-Year Intervention. Health Serv Res. 2015;50(6):1891. 25787874 10.1111/1475-6773.12300 PMC4693854

[11] Graves AJ, Kozhimannil KB, Kleinman KP, Wharam JF. The Association between High-Deductible Health Plan Transition and Contraception and Birth Rates. Health Serv Res. 2016;51(1):187–204. 26118959 10.1111/1475-6773.12326 PMC4722206

[12] Harder T, Takla A, Rehfuess E, Sánchez-Vivar A, Matysiak-Klose D, Eckmanns T, Evidence-based decision-making in infectious diseases epidemiology, prevention and control: matching research questions to study designs and quality appraisal tools. BMC Med Res Methodol. 2014;14(1):69. 24886571 10.1186/1471-2288-14-69 PMC4063433

[13] Turner SL, Karahalios A, Forbes AB, Taljaard M, Grimshaw JM, McKenzie JE. Comparison of six statistical methods for interrupted time series studies: empirical evaluation of 190 published series. BMC Med Res Methodol. 2021;21(1):134. 34174809 10.1186/s12874-021-01306-w PMC8235830

[14] Schaffer AL, Dobbins TA, Pearson SA. Interrupted time series analysis using autoregressive integrated moving average (ARIMA) models: a guide for evaluating large-scale health interventions. BMC Med Res Methodol. 2021;21(1):1–12. 33752604 10.1186/s12874-021-01235-8 PMC7986567

[15] Saeed S, Moodie EE, Strumpf EC, Klein MB. Segmented generalized mixed effect models to evaluate health outcomes. Int J Public Health. 2018;63:547–51. 29549396 10.1007/s00038-018-1091-9

[16] French B, Heagerty PJ. Analysis of longitudinal data to evaluate a policy change. Stat Med. 2008;27(24):5005–25. 18618416 10.1002/sim.3340 PMC3415557

[17] Quandt RE. The estimation of the parameters of a linear regression system obeying two separate regimes. J Am Stat Assoc. 1958;53(284):873–80.

[18] Seber GA, Wild CJ. Nonlinear regression. hoboken. Volume 62. New Jersey: Wiley; 2003. p. 1238. 63.

[19] Simonton DK. Cross-sectional time-series experiments: Some suggested statistical analyses. Psychol Bull. 1977;84(3):489.

[20] Xiao H, Augusto O, Wagenaar BH. Reflection on modern methods: a common error in the segmented regression parameterization of interrupted time-series analyses. Int J Epidemiol. 2020;50(3):1011–5. 10.1093/ije/dyaa148 PMC8271192 33097937

[21] Penfold RB, Zhang F. Use of interrupted time series analysis in evaluating health care quality improvements. Acad Pediatr. 2013;13(6):38–44. 10.1016/j.acap.2013.08.002 24268083

[22] Lopez Bernal J, Cummins S, Gasparrini A. The use of controls in interrupted time series studies of public health interventions. Int J Epidemiol. 2018;47(6):2082–93. 29982445 10.1093/ije/dyy135

[23] Cruz M, Bender M, Ombao H. A robust interrupted time series model for analyzing complex health care intervention data. Stat Med. 2017;36(29):4660–76. 28850683 10.1002/sim.7443

[24] Linden A. Conducting interrupted time-series analysis for single-and multiple-group comparisons. Stata J. 2015;15(2):480–500.

[25] Linden A, Adams JL. Applying a propensity score-based weighting model to interrupted time series data: improving causal inference in programme evaluation. J Eval Clin Pract. 2011;17(6):1231–8. 20973870 10.1111/j.1365-2753.2010.01504.x

[26] Ansari F, Gray K, Nathwani D, Phillips G, Ogston S, Ramsay C, Outcomes of an intervention to improve hospital antibiotic prescribing: interrupted time series with segmented regression analysis. J Antimicrob Chemother. 2003;52(5):842–8. 14563900 10.1093/jac/dkg459

[27] Handley MA, Lyles CR, McCulloch C, Cattamanchi A. Selecting and improving quasi-experimental designs in effectiveness and implementation research. Annu Rev Public Health. 2018;39:5–25. 29328873 10.1146/annurev-publhealth-040617-014128 PMC8011057

[28] Xiao H, Augusto O, Wagenaar BH. Reflection on modern methods: a common error in the segmented regression parameterization of interrupted time-series analyses. Int J Epidemiol. 2021;50(3):1011–5. 33097937 10.1093/ije/dyaa148 PMC8271192

[29] Barone-Adesi F, Gasparrini A, Vizzini L, Merletti F, Richiardi L. Effects of Italian smoking regulation on rates of hospital admission for acute coronary events: a country-wide study. PLoS ONE. 2011;6(3):e17419. 21399685 10.1371/journal.pone.0017419 PMC3047543

[30] Taillie LS, Reyes M, Colchero MA, Popkin B, Corvalán C. An evaluation of Chile’s Law of Food Labeling and Advertising on sugar-sweetened beverage purchases from 2015 to 2017: A before-and-after study. PLoS Med. 2020;17(2):e1003015. 32045424 10.1371/journal.pmed.1003015 PMC7012389

[31] Dorward J, Khubone T, Gate K, Ngobese H, Sookrajh Y, Mkhize S, The impact of the COVID-19 lockdown on HIV care in 65 South African primary care clinics: an interrupted time series analysis. Lancet HIV. 2021;8(3):e158–65. 33549166 10.1016/S2352-3018(20)30359-3 PMC8011055

[32] Angoulvant F, Ouldali N, Yang DD, Filser M, Gajdos V, Rybak A, Coronavirus disease 2019 pandemic: impact caused by school closure and national lockdown on pediatric visits and admissions for viral and nonviral infections-a time series analysis. Clin Infect Dis. 2021;72(2):319–22. 33501967 10.1093/cid/ciaa710 PMC7314162

[33] Leske S, Kõlves K, Crompton D, Arensman E, De Leo D. Real-time suicide mortality data from police reports in Queensland, Australia, during the COVID-19 pandemic: an interrupted time-series analysis. Lancet Psychiatry. 2021;8(1):58–63. 33212023 10.1016/S2215-0366(20)30435-1 PMC7836943

[34] Moore LD, Robbins G, Quinn J, Arbogast JW. The impact of COVID-19 pandemic on hand hygiene performance in hospitals. Am J Infect Control. 2021;49(1):30–3. 32818577 10.1016/j.ajic.2020.08.021 PMC7434409

[35] Ouldali N, Pouletty M, Mariani P, Beyler C, Blachier A, Bonacorsi S, Emergence of Kawasaki disease related to SARS-CoV-2 infection in an epicentre of the French COVID-19 epidemic: a timeseries analysis. Lancet Child Adolesc Health. 2020;4(9):662–8. 32622376 10.1016/S2352-4642(20)30175-9 PMC7332278

[36] Spencer K, Jones CM, Girdler R, Roe C, Sharpe M, Lawton S, The impact of the COVID-19 pandemic on radiotherapy services in England, UK: a population-based study. Lancet Oncol. 2021;22(3):309–20. 33493433 10.1016/S1470-2045(20)30743-9 PMC7825861

[37] Chang DW, Neville TH, Parrish J, Ewing L, Rico C, Jara L, Evaluation of time-limited trials among critically ill patients with advanced medical illnesses and reduction of nonbeneficial ICU treatments. JAMA Intern Med. 2021;181(6):786–94. 33843946 10.1001/jamainternmed.2021.1000 PMC8042568

[38] Manthey J, Jasilionis D, Jiang H, Meščeriakova O, Petkevičienè J, Radišauskas R, The impact of alcohol taxation increase on all-cause mortality inequalities in Lithuania: an interrupted time series analysis. BMC Med. 2023;21(1):22. 36647069 10.1186/s12916-022-02721-6 PMC9841962

[39] Chen Y, Ji X, Xiao H, Unger JM, Cai Y, Mao Z, Impact of the pilot volume-based Drug Purchasing Policy in China: interrupted time-series analysis with controls. Front Pharmacol. 2021;12:804237. 35815118 10.3389/fphar.2021.804237 PMC9262040

[40] Tobías A. Evaluation of the lockdowns for the SARS-CoV-2 epidemic in Italy and Spain after one month follow up. Sci Total Environ. 2020;725:138539. 32304973 10.1016/j.scitotenv.2020.138539 PMC7195141

